# Neonatal handling decreases unconditioned anxiety, conditioned fear, and improves two-way avoidance acquisition: a study with the inbred Roman high (RHA-I)- and low-avoidance (RLA-I) rats of both sexes

**DOI:** 10.3389/fnbeh.2015.00174

**Published:** 2015-07-10

**Authors:** Cristóbal Río-Ȧlamos, Ignasi Oliveras, Toni Cañete, Gloria Blázquez, Esther Martínez-Membrives, Adolf Tobeña, Alberto Fernández-Teruel

**Affiliations:** Medical Psychology Unit, Department of Psychiatry and Forensic Medicine, School of Medicine, Institute of Neurosciences, Autonomous University of BarcelonaBarcelona, Spain

**Keywords:** neonatal handling, anxiety, inbred roman rats, two-way avoidance acquisition, coping style

## Abstract

The present study evaluated the long-lasting effects of neonatal handling (NH; administered during the first 21 days of life) on unlearned and learned anxiety-related responses in inbred Roman High- (RHA-I) and Low-avoidance (RLA-I) rats. To this aim, untreated and neonatally-handled RHA-I and RLA-I rats of both sexes were tested in the following tests/tasks: a novel object exploration (NOE) test, the elevated zero maze (ZM) test, a “baseline acoustic startle” (BAS) test, a “context-conditioned fear” (CCF) test and the acquisition of two-way active—shuttle box—avoidance (SHAV). RLA-I rats showed higher unconditioned (novel object exploration test -“NOE”-, elevated zero maze test -“ZM”-, BAS), and conditioned (CCF, SHAV) anxiety. NH increased exploration of the novel object in the NOE test as well as exploration of the open sections of the ZM test in both rat strains and sexes, although the effects were relatively more marked in the (high anxious) RLA-I strain and in females. NH did not affect BAS, but reduced CCF in both strains and sexes, and improved shuttle box avoidance acquisition especially in RLA-I (and particularly in females) and in female RHA-I rats. These are completely novel findings, which indicate that even some genetically-based anxiety/fear-related phenotypes can be significantly modulated by previous environmental experiences such as the NH manipulation.

## Introduction

Neonatal handling (NH), typically administered to rodents during the first 3 weeks of life, is an environmental treatment that has often been used to study behavioral and neurobiological plasticity. The effects of this manipulation are well documented since the 1950s, when Seymour Levine provided the first demonstration that NH induced an enduring improvement in the ability of rats to learn a two-way active avoidance task (Levine, 1956, 1957). These results have been confirmed by many studies showing that the improving effects of NH extend to a wide variety of tests/tasks and to different strains/lines of rats (and mice) with remarkable long lasting effects. Thus, a large amount of studies have shown that NH increases activity and specific exploratory behavior in rodents, in a variety of unconditioned anxiety/emotionality tests involving different degrees of novelty (e.g., Bodnoff et al., 1987; Escorihuela et al., 1994; Ferré et al., 1995a; Núñez et al., 1995, 1996; McIntosh et al., 1999; Fernández-Teruel et al., 2002a; Cañete et al., 2015), although absence of effects, task-specific effects, and sex-specific effects have also been reported (see review by Raineki et al., 2014). In addition, concerning its effects on conditioned fear/anxiety-related measures, studies with unselected rats have shown that NH enduringly reduces conflict-induced lick suppression and conditioned freezing (Núñez et al., 1996), accelerates two-way active avoidance acquisition (Escorihuela et al., 1992, 1994; Núñez et al., 1995), thus replicating and extending the original Levine's findings, and reduces learned helplessness (Tejedor-Real et al., 1998). NH has also been reported to decrease stress-induced corticosterone, ACTH, and prolactin secretion (e.g., Levine, 1957; Meaney et al., 1988, 1991; Núñez et al., 1996; Anisman et al., 1998; Raineki et al., 2014). Thus, from a behavioral and neuroendocrine perspective, NH-treated rodents appear to have an improved ability to adapt, or to efficiently cope with challenging/stressful environmental conditions. Finally, NH manipulation generally improves cognition in rats and mice under different spatial learning/memory paradigms, although such effects are strain- and sex-dependent (e.g., Wilson and Jamieson, 1968; Meaney et al., 1988; Zaharia et al., 1996; Fernández-Teruel et al., 2002a; Stamatakis et al., 2008; Raineki et al., 2014; Cañete et al., 2015). However, in the cognitive (learning and memory) domain there are controversial results, as with the exception of shuttle box avoidance acquisition (see refs. above), the NH procedure generally impairs aversive learning in several tasks (see review by Raineki et al., 2014).

One of the most validated genetic rat models for the study of fear/anxiety- and stress-related phenotypes is constituted by the Roman High- and Low-avoidance (RHA and RLA, respectively) rat lines/strains. They were initially selected and bred on the basis of their very good (RHA) vs. extremely poor (RLA) acquisition of the two-way active—shuttle box—avoidance response (Bignami, 1965; Driscoll and Bättig, 1982; Driscoll et al., 1998). Two inbred strains (RHA-I and RLA-I) derived from the original outbred (RHA/Verh and RLA/Verh) lines, are maintained at the Autonomous University of Barcelona since 1997 (Escorihuela et al., 1999; Driscoll et al., 2009), while colonies of the outbred RHA/RLA rat lines are maintained at Geneva (Switzerland; Dr. Steimer; e.g., Steimer and Driscoll, 2005) and Cagliari (Italy; Prof. Giorgi and Corda; e.g., Giorgi et al., 2007).

Learning a two-way avoidance task in a shuttle box involves a “passive avoidance/active avoidance” conflict during the initial stages of acquisition (i.e., a tendency to freeze–receiving the electric shock- runs against a tendency to actively cross to the opposite compartment -avoiding the insult-) which is mediated by anxiety (e.g., Wilcock and Fulker, 1973; Gray, 1982; Gray and McNaughton, 2000; Vicens-Costa et al., 2011). Accordingly, shuttle box avoidance acquisition has been shown to be inversely related to anxiety/fear (e.g., Weiss et al., 1968; Gray, 1982; Fernández-Teruel et al., 1991a,b; Escorihuela et al., 1993; Gray and McNaughton, 2000; López-Aumatell et al., 2009a,b, 2011; Vicens-Costa et al., 2011; Díaz-Morán et al., 2012). Not surprisingly, therefore, the extensive research conducted with the RLA and RHA rats over near four decades has led to the conclusion that anxiety/fearfulness and stress sensitivity are among the most prominent behavioral traits separating the two lines/strains. In fact, RLAs (both from the outbred lines and from the inbred strain) are more anxious and/or fearful than their RHA counterparts in a wide series of unconditioned and conditioned tests/tasks (e.g., Ferré et al., 1995b; Escorihuela et al., 1999; Steimer and Driscoll, 2003, 2005; Driscoll et al., 2009; López-Aumatell et al., 2009a,b; Díaz-Morán et al., 2012; Martinez-Membrives et al., 2015). Moreover, RLA rats display enhanced frustration responses following reward down-shift (e.g., Torres et al., 2005; Rosas et al., 2007; Sabariego et al., 2013) and higher stress-induced HPA-axis and prolactin responses than RHAs (e.g., Steimer and Driscoll, 2003, 2005; Carrasco et al., 2008; Díaz-Morán et al., 2012). To sum up, it is commonly accepted that, compared with RHAs, RLAs rats display increased anxiety, fearfulness, stress sensitivity, and a predominantly passive (reactive) coping style when facing situations involving conflict (e.g., Steimer and Driscoll, 2003, 2005; Díaz-Morán et al., 2012).

As mentioned earlier, NH procedure generally appears to improve the subjects' ability to adapt to, or to efficiently cope with conflicting and/or stressful conditions. However, most of the research on NH effects has been performed in one gender, usually male rats or mice. Interactions between NH and sex have been observed in some reports which evaluated NH effects in unselected rats of both sexes. To say just a few examples (see also “Discussion”): NH improved spatial learning (in the Morris Water Maze; MWM) only in males (Stamatakis et al., 2008) while, in different studies, spatial learning in the “Y” maze was improved by NH in females and impaired in males (Noschang et al., 2012), and long-term retention of inhibitory avoidance was impaired only in females (Kosten et al., 2007). The striking sex differences in the effects of NH tell us that gender must be considered as an important (or even crucial) variable in behavioral and neurobiological studies of NH induced effects and/or mechanisms.

Thus, the present study was aimed to evaluate whether the NH procedure is able to improve coping ability in both inbred Roman strains and sexes, with an especial focus on RLA-I rats. If so, we would expect that handled RLA-I rats present a more active coping style than untreated RLA-I animals, which would be reflected by unlearned and/or learned anxiety/fear measures. To this aim, non-handled (undisturbed) and NH treated inbred Roman Low- (RLA-I) and High-avoidance (RHA-I) rats of both sexes were evaluated in a test battery devoted to measure several types of unconditioned and conditioned anxiety/fear-related responses: a “novel object exploration” (NOE) test, the elevated zero-maze (ZM), a baseline acoustic startle response test (BAS), a context-conditioned fear (CCF) test and the acquisition of the two-way active avoidance (SHAV) task. This represents the first time that the effects of NH on both unconditioned and conditioned anxiety/fear (including shuttle box avoidance acquisition) are evaluated in “inbred” Roman rats from both strains and sexes.

## Materials and methods

### Animals

Pregnant inbred Roman High- (RHA-I) and Low-Avoidance (RLA-I) rats from our permanent colony at the Autonomous University of Barcelona (Medical Psychology Unit, Department Psychiatry and Forensic Medicine) were used in the present study. They were individually housed and were maintained with food and water freely available, with a 12-h light-dark cycle (light on 0800 h) and controlled temperature (22 ± 2°C). They were randomly distributed across the following experimental groups to which their offspring would be assigned: control animals, which were not disturbed until weaning (C), and animals that received neonatal handling (NH, see procedure below). All care was taken to avoid litter effects, by using a sufficiently large number of litters per group. Thus, each experimental group contained animals from at least 6 different litters. At postnatal day 1, litters were culled to a maximum of 12 pups (without any compensation for the number of males or females). After weaning (postnatal day 21st) the pups were housed in pairs of the same litter, sex and group in standard macrolon cages (50 × 25 × 14 cm) under the above conditions. Experiments were performed using 50 RLA-I and 29 RHA-I rats from the 59th generation of inbreeding. At the beginning of the experiments subjects were 2 months old (weight, 167 ± 20 g; mean ± *SD*; see Table 1 for details of the sample). Experiments were performed during the light cycle, between 09:00 and 19:00 h in accordance with the Spanish legislation on “Protection of Animals Used for Experimental and Other Scientific Purposes” and the European Communities Council Directive (86/609/EEC) on this subject.

**Table 1:** **Animal samples and experimental goups**.

**Strain**	**Treatment group**	**Sex**	**Sample**
RLA-I	Control (C)	♂	12 (final *n* = 9^*^
	Handled (NH)	♂	17
	Control (C)	♀	12
	Handled (NH)	♀	12
RHA-I	Control (C)	♂	7 (final *n* = 6)^*^
	Handled (NH)	♂	8
	Control (C)	♀	7
	Handled (NH)	♀	8

* *Final n = 9 and n = 6 in RLA-I and RHA-I control groups because of technical problems in several tests/tasks*.

## Procedure and apparatus

### Neonatal handling (NH)

NH was given twice daily between postnatal days 1 and 21 (see Fernández-Teruel et al., 1992; Escorihuela et al., 1995; Steimer et al., 1998). The first daily handling session, administered in the morning (approximately between 9:30 and 10:30 h a.m.), consisted of first removing the mother from the litter and then placing the pups gently and individually in plastic cages (35 × 15 × 25 cm) lined with paper towel for a total period of 8 min. After 4 min in this situation, each pup was individually (and gently) handled and stroked for 3–4 s and returned to the same cage for the remaining 4 min. At the end of the 8-min period, each pup was gently handled for another 3–4 s and then returned to its homecage. When all the pups from one litter were back in their homecage, the mother was returned to it. The same procedure was conducted in the evening (2nd time; approximately at 5:00 h p.m.). NH was carried out in a room different from the animal room, maintaining the temperature at 24°C. NH finished at postnatal day 21. Weaning was done at postnatal day 21, after finishing the last NH session. Control (C) non-handled groups were left undisturbed, except for regular cage cleaning once a week, until weaning.

### Test 1: novel object exploration test (NOE)

**Figure 1:**
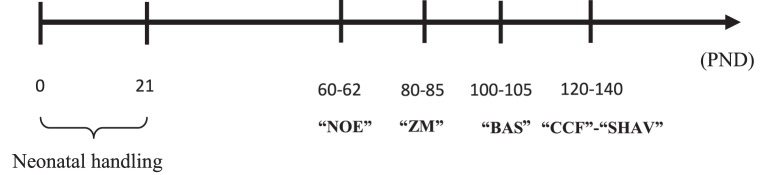
**Abbreviations: NOE, novel object exploration test; ZM, elevated zero-maze test; BAS, baseline acoustic startle; CCF, context-conditioned freezing; SHAV, two-way active –shuttle box- avoidance; PND, postnatal day**.

In order to assess emotional reactivity (or behavioral inhibition under novelty, or “curiosity”) a novel object exploration (NOE) test was conducted. The test consisted of the evaluation of the exploratory response of rats when a novel object was introduced in their home cage. Rats were 60 days-old at the beginning of the NOE test, and they were housed in pairs of the same sex, strain, and treatment condition. The test started by removing the food from the home cage (leaving only four pellets in each cage). One hour later, the novel object (graphite pencil Staedtler Noris, HB n°2) was perpendicularly introduced in their home cages through the grid cover, until it made contact with the cage bedding. To facilitate observation of the rats each individual cage was pulled from the rack about 15 cm, which allowed to score the latency to the first exploration (*LAT-NOE*; time spent until the first exploration of the novel object) and the total time (*Time-NOE*) spent exploring the pencil for each individual rat. The experimenter/observer was standing at 50 cm from the cage front. The NOE test lasted 3 min (see Figure 1).

### Test 2: elevated zero maze (EZM)

The maze, similar to that described by Shepherd et al. (1994) (1) comprised an annular platform (i.e., a circular corridor; 105 cm diameter; 10 cm width) made of black plywood and elevated to 65 cm above the ground level. It had two open sections (quadrants) and two enclosed ones (with walls 40 cm height). The subject (80 days-old) was placed in an enclosed section facing the wall. The apparatus was situated in a black testing room, dimly illuminated with red fluorescent light, and the behavior was videotaped and measured outside the testing room. Time spent in open sections (*ZM-T*), number of entries into open sections (*ZM-E*), and number of episodes of exploratory activity at the edge of the test, namely “head dips” (*ZM-HD*), were measured for 5 min (see López-Aumatell et al., 2008, 2009a; see Figure 1).

### Test 3: baseline acoustic startle response (BAS)

Four sound-attenuated boxes (Sr-Lab Startle Response system, San Diego Inst., San Diego, USA) diffusely illuminated (10 w) were used (90 × 55 × 60 cm). Each box housed a Plexiglas cylinder (8.2 cm in diameter, 25 cm in length) with a grid placed in the bottom, resting on a plastic frame. For any test session each animal was placed in the cylinder, and movements of the cylinder resulting from startle responses were transduced by a piezoelectric accelerometer (Cibertec S.A. Madrid) into a voltage which was amplified, digitized and saved into a computer for analysis. The session started with 5 min of habituation. A white noise generator provided background noise of 55 dB. Then, 25 trials of acoustic startle stimuli of 105 dB and 40 ms of duration were delivered by a loudspeaker, mounted at distance of 23 cm above the plexiglas cylinder. The inter-trial interval (ITI) was 15 s in average (range 10–20 s). Startle response amplitude was defined as the maximum accelerometer voltage during the first 200 ms after the startle stimulus onset (see López-Aumatell et al., 2008; see Figure 1).

### Tests 4 and 5: context conditioned freezing (CCF) and two-way active—shuttle box—avoidance acquisition (SHAV)

The experiment was carried out with two identical shuttle boxes (Letica, Panlab, Barcelona, Spain) each placed within independent sound-attenuating boxes constructed of plywood. A dim and diffuse illumination was provided by a fluorescent bulb placed behind the opaque wall of the shuttle boxes. The experimental room was kept dark. The shuttle boxes consisted of two equally sized compartments (25 × 25 × 28 cm), connected by an opening (8 × 10 cm). Training consisted of a single 50-trial session for the RHA-I strain, and two 50-trial sessions, spaced 24 h apart, for RLA-I rats. RLA-I rats were trained twice as much as RHA-I rats because we did not expect any NH effect on RHA-I rats, due to roof effects (i.e., they usually attain a >60% avoidance response levels in the first 50-trial session). A 2400-Hz, 63-dB tone plus a light (from a small 7-w lamp) functioned as the CS (conditioned stimulus). The US (unconditioned stimulus) which commenced at the end of the CS, was a scrambled electric shock of 0.7 mA delivered through the grid floor. Once the rats were placed into the shuttle box, a 4-min familiarization period (without any stimulus) elapsed before training commenced. Each of the 50 (or 100 -in case of RLA-I rats-) training trials consisted of a 10-s CS, followed by a 20-s US. The CS or US was terminated when the animal crossed to the other compartment, with crossing during the CS being considered as an avoidance response and during the US as an escape response. Once a crossing had been made or the shock (US) discontinued, a 60-s inter-trial interval (ITI) was presented during which crossings (ITC) were scored within each block of trials. Freezing behavior, defined as the complete absence of movements except for breathing, was also scored (by a well-trained observer) during the 60-s inter-trial intervals of trials 2–5 as an index of context-conditioned fear (CCF; during trials 2–5 no rat made any avoidance response, i.e., all rats received electric shock in these trials). The measure of freezing during the inter-trial interval of trial 1 was excluded because it is not a proper measure of context conditioning.

The variables recorded were the number of avoidances (SHAV) and inter-trial crossings (ITCs), either grouped in blocks of 10 trials or accumulated in one (SHAV50, ITC50), or two (SHAV100, ITC100) sessions (e.g., see López-Aumatell et al., 2011; Díaz-Morán et al., 2012; see Figure 1).

## Statistical analysis

Statistical analysis was performed using the “Statistical Package for Social Science” (SPSS, version 17).

Pearson's correlation coefficients were performed among the main variables.

Factorial 2 × 2 × 2 ANOVAs (“2 strain” × “2 treatment conditions” × “2 sex”) were applied to measures from NOE, ZM, and CCF tests, as well as for total measures of the shuttle box avoidance task. Appropriate repeated measures ANOVAs with “5-trial blocks” as within-subject factor were applied to BAS test (“2 strain” × “2 treatment conditions” × “2 sex” × “5 block” ANOVA), and to shuttle box avoidance acquisition with “10-trial blocks” as within-subject factor (“2 strain” × “2 treatment conditions” × “2 sex” × “10 block” ANOVAs).

*Post-hoc* Duncan's multiple range tests were applied to all dependent variables following significant ANOVA effects. A Student's *t*-test (independent samples) was also applied to avoidance results from male “control” and “NH” RLA-I groups, because we had the a priori hypothesis that NH treatment would improve avoidance acquisition in RLA-I rats. Significance level was set at *p* ≤ 0.05.

## Results

### “Novel object exploration” test (NOE)

**Figure 2:**
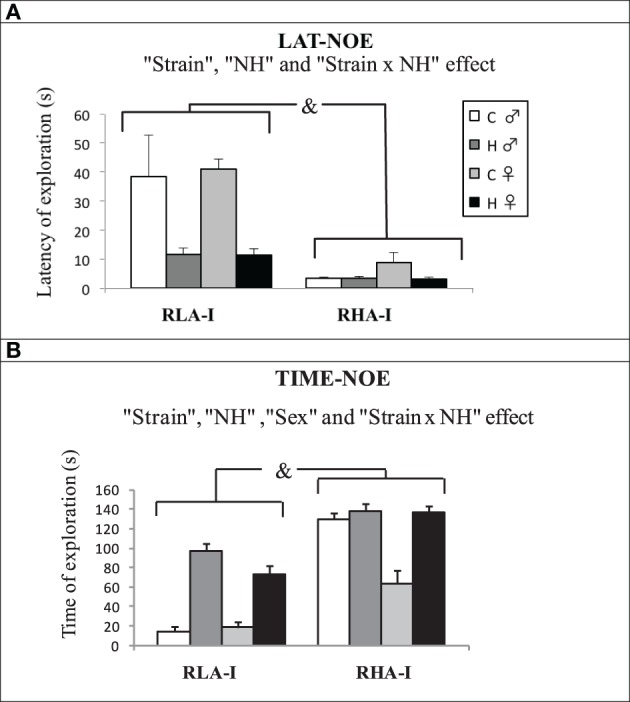
**Mean ± S.E.M of (A) “Latency –time elapsed—until the first exploration of the novel object” and (B) “Total time spent exploring the novel object” in Experiment 1 (NOE test)**. &, indicates the “Strain” effect, *p* < 0.05. Group symbols: C, control non-handled group; H, neonatally handled (NH) group.

The results of the NOE test (Figures 2A,B) showed that, compared to RHA-I rats, RLA-I animals presented higher latency (to explore for the first time the novel object; LAT-NOE) and less time spent exploring the novel object (TIME-NOE) [“Strain” effect on both parameters, *F*_(1, 78)_ = 17.36, *p* < 0.001, and *F*_(1,78)_ = 118.30, *P* < 0.001, respectively]. As expected, NH significantly reduced LAT-NOE and increased TIME-NOE in both rat strains [“NH” effect, *F*_(1,78)_ = 9.66, *p* ≤ 0.003, and *F*_(1,78)_ = 80.40 *P* < 0.001, respectively]. A “sex” effect was found only on TIME-NOE [*F*_(1, 78)_ = 13.08, *p* = 0.001], indicating that females (particularly RHA-Is) spent overall less time exploring the novel object compared to males (Figure 2B). There were also “Strain × NH” interactions for LAT-NOE and TIME-NOE [*F*_(1, 78)_ = 6.37, *p* ≤ 0.01, and *F*_(1,78)_ = 5.32 *P* = 0.02, respectively], as NH effects were globally stronger in RLA-I rats of both sexes.

### “Elevated zero maze” test (ZM)

**Figure 3:**
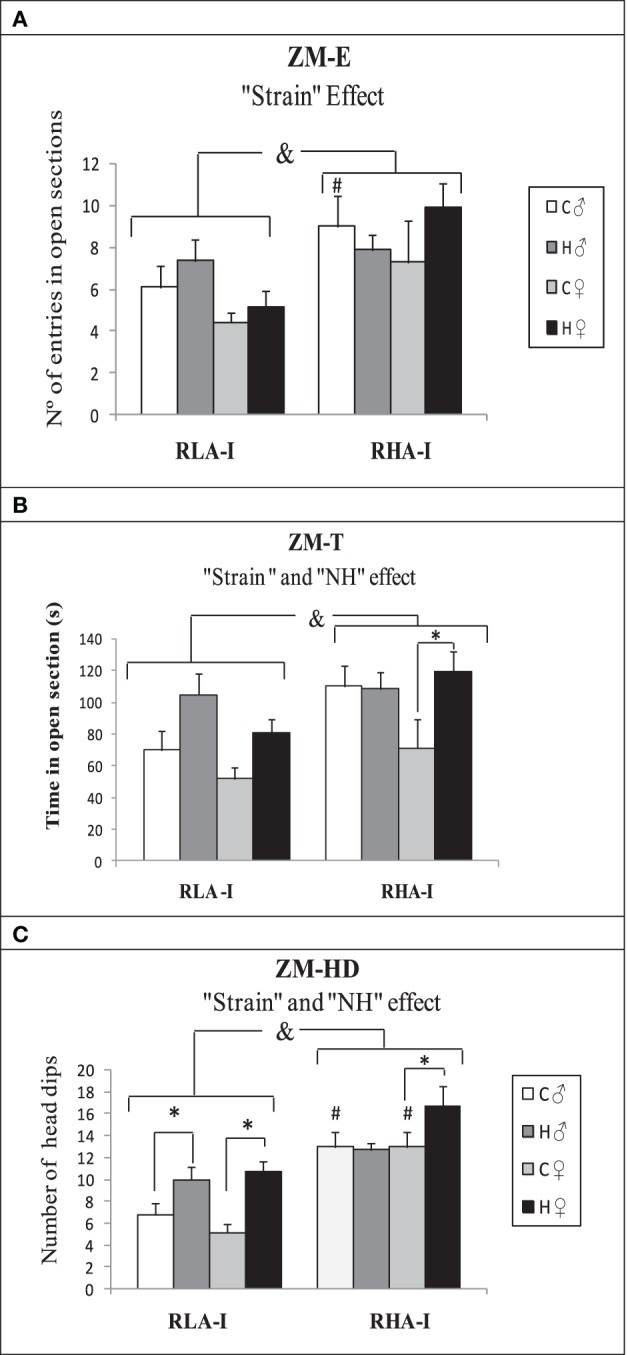
**Mean ± S.E.M. of (A) “Number of entries (ZM-E),” (B) “Time spent in open sections (ZM-T)” and (C) “Number of head dips (ZM-HD)” in Experiment 2 (“Elevated zero maze” test,” ZM)**. &, indicates the “Strain” effect (see text for significance); ^*^p < 0.05 between the groups indicated (Duncan's multiple range tests following significant ANOVA effects); #*p* < 0.05 vs. respective control (C) group of the RLA-I strain (Duncan's multiple range tests following significant ANOVA effects). Group symbols: C, control non-handled group; H, neonatally handled (NH) group.

The results of the ZM test (Figures 3A–C) showed “Strain” effects on ZM-E [*F*_(1, 78)_ = 12.13, *p* ≤ 0.001], ZM-T [*F*_(1, 78)_ = 7.29, *p* ≤ 0.009] and ZM-HD [*Fs*_(1, 78)_ = 41.55, *p* < 0.001], with RHA-I rats showing overall higher scores in the three parameters (Figures 3A–C). “NH” effects were found in ZM-T [*F*_(1, 78)_ = 8.60, *p* ≤ 0.005] and in ZM-HD [*F*_(1, 78)_ = 11.85, *p* ≤ 0.001], reflecting that neonatally-handled groups globally spent more time in open sections and performed more head dips than untreated animals (Figures 3B,C; see also Duncan's tests in Figures 3B,C).

### “Baseline acoustic startle response” test (BAS)

**Figure 4:**
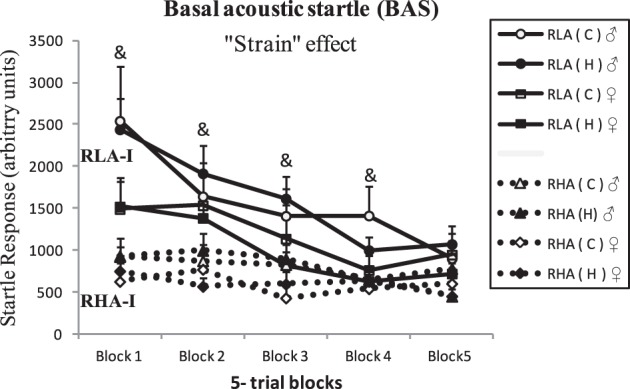
**Mean ± S.E.M of baseline acoustic startle response (BAS, in arbitrary units) across 5-trial blocks (Block1–Block5)**. &, significant (*p* < 0.05) “Strain” effects (factorial 2 × 2 × 2 ANOVAs) in these blocks. The overall repeated measures ANOVA also yielded a significant “Strain” effect, as indicated in the figure (*p* < 0.001, see text for more details). Group symbols: C, control non-handled group; H, neonatally handled (NH) group.

Figure 4 shows the results of the BAS test. The repeated measures ANOVA (“2 strain” × “2 treatment conditions” × “2 sex” × “5 blocks of trials”) indicated a “strain” effect, as taking the session as a whole, the RLA-I strain displayed higher acoustic startle response than the RHA-I strain [“Strain” effect, *F*_(1, 71)_ = 12.26, *p* ≤ 0.001]. ANOVA also showed significant “Block” and “Block × Strain” effects [*F*_(3, 222)_ > 22.22, *p* < 0.001, and *F*_(3, 222)_ = 9.11, *p* < 0.001, respectively], indicating both an habituation effect (on both strains) as well as that such a habituation is relatively more marked in RLA-I rats (Figure 4). Further One-Way ANOVAs per each 5-trial block showed between-strain differences (i.e., overall higher BAS scores in RLA-I than RHA-I rats) in all blocks except in the last one [Block1–Block4, all *Fs*_(7, 78)_ > 2.10, all *p* ≤ 0.05; see Figure 4]. No NH effect was observed.

### “Context-conditioned freezing” (“CCF”) and “two-way active—shuttle box—avoidance acquisition” test (“SHAV”)

**Figure 5:**
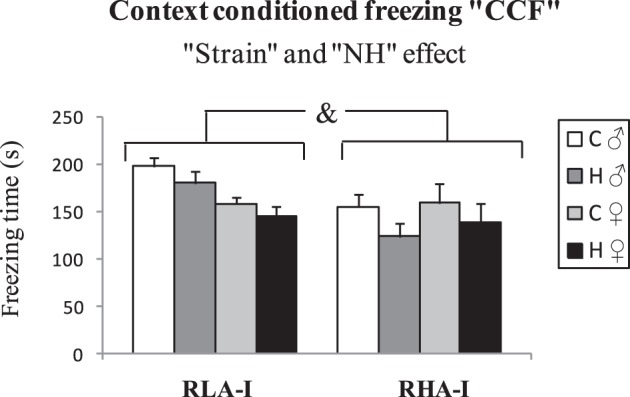
**Means ± S.E.M. of “Context conditioned freezing (CCF) in Experiment 4 (“The two-way active shuttle box avoidance acquisition”)**. &, indicates the “Strain” effect (see text for significance). Group symbols: C, control non-handled group; H, neonatally handled (NH) group.

Results of the “context conditioned freezing” (CCF) test are shown in Figure 5. One-Way ANOVA showed a global “Strain” effect, with the RLA-I groups performing more freezing behavior than the RHA-I strain [“Strain” effect, *F*_(1, 76)_ = 6.79, *p* ≤ 0.011, Figure 5]. Interestingly, a global “NH” effect was also present, as NH decreased the time spent freezing in both strains [“NH” effect *F*_(1, 76)_ = 4.11, *p* = 0.046; Figure 5]. There was also a “Strain × Sex” effect, mainly because there was a trend for RLA-I female groups to show lesser freezing than their respective male groups, and that tendency was not present in RHA-I rats [“Strain × Sex” effect, *F*_(1, 76)_ = 5.39, *p* = 0.023; Figure 5].

**Figure 6:**
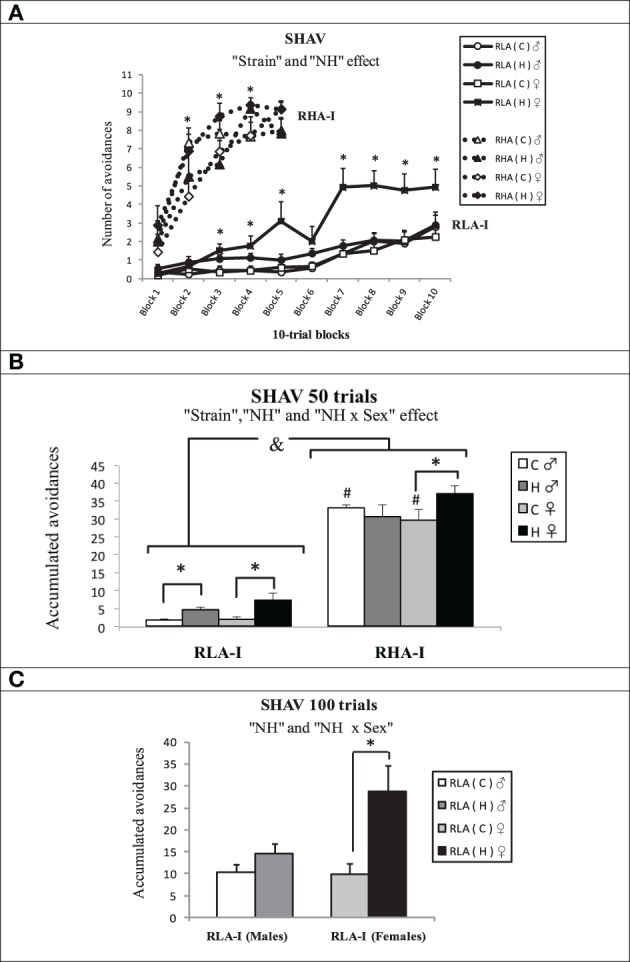
**Mean ± S.E.M. of (A) “Avoidances” in RHA-I (50 trials) and RLA-I (100 trials) grouped in blocks of 10 trials each; (B) “Accumulated avoidances” in the first 50-trial session for both rat strains; (C) “Accumulated avoidances” for the RLA-I strain in the total 100 shuttle box acquisition trials. (A)** ^*^p < 0.05 vs. the respective control (C) group of the same sex (Duncan's tests after significant ANOVA effects). **(B,C)** &, indicates the “Strain” effect (see text for significance); ^*^p < 0.05 between the groups indicated (Duncan's tests following significant ANOVA effects); #*p* < 0.05 vs. respective control (C) group of the RLA-I strain (Duncan's tests following significant ANOVA effects). Group symbols: C, control non-handled group; H, neonatally handled (NH) group.

Figures 6A–C shows the results of two-way avoidance (SHAV) acquisition. The repeated measures ANOVA applied to results from the first 50-trial session (“2 Strain” × “Treatment conditions” × “2 Sex” × “5 blocks of 10 trials”) showed that RHA-I performed more avoidance responses than RLA-I rats [“Strain” effect, *F*_(1, 76)_ = 462.7, *p* < 0.001; Figures 6A,B] and also a global “NH” effect [*F*_(1, 76)_ = 6.10, *p* = 0.016; Figures 6A,B], with neonatally-handled animals performing overall more avoidances than untreated/control rats (Figures 6A,B). Duncan's test showed statistical differences between control and handled RHA-I females (in several 10-trial blocks—Figure 6A as well as in the whole 50-trial session—Figure 6B) as well as between control and handled RLA-I females (in several 10-trial blocks—Figure 6A and in the whole 50-trial session—Figure 6B), while a Student's *t*-test for independent samples showed differences between control and handled RLA-I males in the whole 50-trial session [*t*_(24)_ = 2.68, *p* = 0.014; Figure 6B. This *t*-test was applied because we had the—directed– a priori hypothesis that NH procedure would improve avoidance acquisition in RLA-I rats].

The repeated measures ANOVA of the first 50-trial session also showed “Block” and “Block × Strain” effects [both ANOVAs, *Fs*_(4, 69)_ > 93.96, *p* ≤ 0.001; Figure 6A], thus respectively reflecting (i) the overall significant learning curves as well as (ii) that RHA-I rats learned much faster than RLA-I rats. There was also a “NH × Sex” effect [*F*_(1, 76)_ = 5.24, *p* = 0.025; Figure 6B], mainly because NH induced positive effects on avoidance acquisition of all groups except RHA-I males (Figure 6B).

Analysis of the whole 100 acquisition trials (i.e., the two training sessions) in RLA-I groups (repeated measures ANOVA, “2 treatment conditions” × “2 sex” × “10 blocks of 10 trials” as within-subject factor; SHAV100 trials in Figure 6C) showed a “NH” effect [*F*_(1, 46)_ = 10.68, *p* = 0.002; Figures 6A,C], with handled animals performing overall better than control rats (Figure 6C), and a “NH × Sex” effect [*F*_(1, 46)_ = 4.24, *p* = 0.045], as NH more markedly increased the number of avoidances in RLA-I females (see Duncan's test in Figure 6C) than in males. ANOVA also showed “Block,” “Block × NH,” and “Block × Sex” effects [all *Fs*_(6, 266.11)_ > 2.18, *P* ≤ 0.05](Figure 6A), thus respectively indicating that (i) RLA-I rats show a significant acquisition curve along the 100 training trials, (ii) such an acquisition curve depends on the treatment condition (as NH-induced acquisition improvements are different depending on which 10-trial block is taken into account), and (iii) such an acquisition curve depends on the gender (particularly because of the pronounced NH effect on females, across different 10-trial blocks, which is not present in RLA-I males) (see Figure 6A).

**Figure 7:**
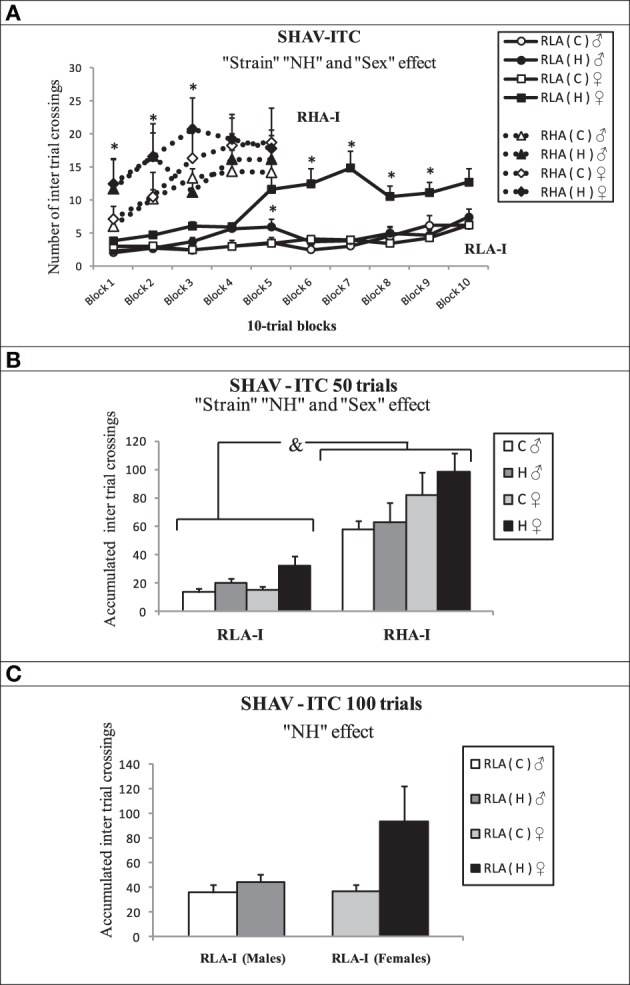
**Mean ± S.E.M. in (A) “Inter trial crossings (ITC)” in RHA-I (50 trials) and RLA-I (100 trials) grouped in blocks of 10 trials each. (B)** “Accumulated Inter trial crossings” in the first 50-trial session for both strains; **(C)** “Accumulated Inter trial crossings” for the RLA-I strain in the total 100 shuttle box acquisition trials. **(A)** ^*^p < 0.05 vs. the respective control **(C)** group of the same sex (Duncan's tests after significant ANOVA effects). **(B)** &, indicates the “Strain” effect (see text for significance); ^*^p < 0.05 between the groups indicated (Duncan's tests following significant ANOVA effects). Group symbols: C, control non-handled group; H, neonatally handled (NH) group.

Figures 7A,B shows ITCs (inter-trial crossings) results during avoidance acquisition training. The repeated measures ANOVA applied to results from the first 50-trial session (“2 Strain” × “Treatment conditions” × “2 Sex” × “5 blocks of 10 trials”) showed that RHA-I performed more ITCs than RLA-I rats [“Strain” effect, *F*_(1, 76)_ = 96.4, *p* < 0.001; Figures 7A,B], a global “NH” effect [*F*_(1, 76)_ = 5.3, *p* = 0.024; Figures 6A,B], with neonatally-handled animals performing overall more ITCs than untreated rats (Figures 7A,B), and a “Sex” effect [*F*_(1, 76)_ = 7.5, *p* = 0.008] indicating that females of both strains performed more ITCs than male rats (Figures 7A,B). Similar to SHAV50 results, there were also “Block” and “Block × Strain,” as well as “Block × Sex” effects on ITCs [for all parameters, *Fs*_(4, 252.63)_ > 2.89, *p* ≤ 0.03].

Analysis of ITCs along the whole 100 training trials (repeated measures ANOVA, “2 treatment conditions” × “2 sex” × “10 blocks of 10 trials” as within-subject factor; SHAV-ITC 100; Figure 7C), only in the RLA-I groups, showed a NH effect [*F*_(1, 46)_ = 4.75, *p* = 0.035], as neonatally-handled animals performed more ITCs than untreated ones (see Figure 7C). There was also a “Block” effect [*F*_(3, 122.06)_ = 5.96, *p* = 0.001] (Figure 7A), reflecting the overall ascending progression of ITCs across successive 10-trial blocks.

### Correlations among variables

Pearson correlations are shown in Table 2. The most relevant trends to highlight are between-test correlations. In this regard, significant correlations are observed between ZM and NOE variables (from *r* = −0.26 to *r* = 0.53), indicating that both tests might be partly measuring similar anxiety-related traits. There are also low but significant correlations among both NOE and ZM variables with BAS parameters (NOE with BAS variables, from *r* = 0.22 to *r* = 0.28. ZM with BAS variables: *r* = −0.25 between ZM-E and BAS21-25; *r* = −0.29 between ZM-HD and BAS; see Table 2). Most importantly, there were very relevant correlations among ZM variables and SHAV and ITC (ranging from r = 0.29 to r = 0.56; Table 2), as well as between NOE variables and SHAV and ITCs (ranging from *r* = −0.30 to *r* = 0.58; Table 2) and between BAS (acoustic startle) responses and SHAV and ITCs (ranging from *r* = −0.25 to *r* = −0.39; Table 2), thus suggesting that unconditioned anxiety-related trait is negatively associated with two-way avoidance acquisition, i.e., the higher the unconditioned anxiety levels in those three tests the poorer the acquisition levels in the avoidance task.

**Table 2:** **Pearson correlation coefficients are shown**.

	**LAT-NOE**	**TIME-NOE**	**ZM-E**	**ZM-T**	**ZM-HD**	**BAS1_5**	**BAS21_25**	**BAS**	**CCF**	**SHAV1**	**ITC1**	SHAV2 (^*^)	ITC2 (^*^)	TOTAL-SHAV (^*^)
LAT-NOE	1													
TIME-NOE	−,60^***^	1												
ZM-E	−,26^*^	,32^**^	1											
ZM-T	−,30^**^	,41^***^	,82^***^	1										
ZM-HD	−,33^**^	,53^***^	,51^***^	,59^***^	1									
BAS1_5	,22^*^	−,19	−,06	−,03	−,33^**^	1								
BAS21_25	,26^*^	−,11	−,25^*^	−,20	−,19	,67^***^	1							
BAS	,28^*^	−,17	−,15	−,10	−,29^**^	,92^***^	,87^***^	1						
CCF	,14	−,18	−,11	−,06	−,20	,17	,13	,17	1					
SHAV1	−,39^***^	,58^***^	,34^**^	,25^*^	,56^***^	−,39^***^	−,25^*^	−,36^**^	−,40^***^	1				
ITC1	−,30^**^	,45^***^	,29^**^	,21	,50^***^	−,30^**^	−,17	−,27^*^	−,40^***^	,88^***^	1			
SHAV2 (^*^)	−,11	,17	−,04	,03	,20	−,13	−,13	−,16	−,36^*^	,75^***^	,66^***^	1		
ITC2 (^*^)	−,11	,15	−,02	,05	,29^*^	−,11	−,07	−,12	−,37^**^	,72^***^	,78^***^	,74^***^	1	
TOTAL-SHAV (^*^)	−,17	,23	−,05	,02	,20	−,10	−,13	−,14	−,40^**^	,88^***^	,74^***^	,98^***^	,77^***^	1

*Significant values are in bold*.

* p < 0.05;

** p < 0.01;

*** *p < 0.001 (two-tailed)*.

(*) *Refers to the RLA-I groups only (thus n = 50), which were the only groups performing 100 trials in the shuttle box avoidance task*.

## Discussion

In the present study we have investigated, for the first time: (1) NH effects in inbred RHA-I/RLA-I rats of both sexes, (2) by using a test battery which included both unconditioned (NOE, ZM, and BAS) anxiety/fear tests and -most importantly- a context-conditioned fear test and shuttle box avoidance acquisition (i.e., the trait which constitutes the basis of genetic selection of RLA-I and RHA-I rats). We have found that, compared with their RHA-I counterparts, RLA-I rats show higher unconditioned anxiety/fear-related responses in the novel object exploration (NOE) and elevated zero-maze (ZM) tests, as well as in the baseline acoustic startle (BAS) test. These results agree with previous reports showing similar differences between the RLA-I and RHA-I strains in a variety of novelty/conflict tests (e.g., Driscoll et al., 2009; López-Aumatell et al., 2009a,b; Díaz-Morán et al., 2012; Martinez-Membrives et al., 2015; see “Introduction” for further references). As expected, and also in agreement with previous reports, RLA-I rats also displayed an overall increase of context-conditioned freezing and markedly impaired acquisition of the two-way active avoidance response compared with RHA-I rats (e.g., López-Aumatell et al., 2009a,b; Díaz-Morán et al., 2012; Martinez-Membrives et al., 2015; see “Introduction”).

The main novel findings of the present study concern the effects of neonatal handling. Thus, regarding the unconditioned tests, i.e., NOE and ZM, we have found that NH increases exploration of both the novel object (NOE) and the open sections of the ZM test in both rat strains, although in the NOE test such effects are apparently more marked in RLA-I rats, which are more behaviorally inhibited (i.e., more anxious) than RHA-I rats in both tests (compare untreated rats of both strains in Figures 2, 3). Actually, the levels attained by NH-treated RLA-I rats in NOE measures tend to approach the response levels of untreated RHA-I rats. These NH effects are overall in agreement with those previously reported on several (unconditioned) novelty/anxiety-related traits in unselected rats (e.g., Escorihuela et al., 1994; Ferré et al., 1995a; Núñez et al., 1995, 1996; Fernández-Teruel et al., 2002a; Raineki et al., 2014; see further references in the “Introduction”) as well as in the Roman rats from the “outbred” lines (e.g., Fernández-Teruel et al., 1992; Steimer et al., 1998).

Importantly, the present study is the first demonstration that NH enduringly improves two-way avoidance acquisition in “inbred” RLA-I rats of both sexes and in female RHA-I rats (see “NH × sex” interactions in “Results”). The positive effect of NH manipulation on avoidances in RLA-I rats is also more pronounced in females, as reflected by significant “NH × sex” effects on SHAV100 (see and Figures 6A–C). NH also induced a significant increase of ITCs, both considering all groups (see Figures 7A,B) or only RLA-I groups (see Figure 7C). We have to remind here that the relevant literature shows that ITCs are positively related with (and are a positive predictor of) two-way avoidance acquisition, i.e., ITCs are “pseudoavoidance” responses indicating that animals are developing active coping strategies to solve the “passive avoidance/active avoidance” conflict involved in the task (for review see Castanon et al., 1995; Aguilar et al., 2004), as it is also suggested by the positive SHAV-ITC correlations observed here (see Table 2). In parallel to these results, NH overall decreased context-conditioned freezing (i.e., classically conditioned fear) in both rat strains. Fear to the context during the initial stages of shuttle box avoidance training is known to be inversely related to effective avoidance acquisition (e.g., López-Aumatell et al., 2011; Vicens-Costa et al., 2011; Díaz-Morán et al., 2012; Martinez-Membrives et al., 2015). The negative correlations between context-conditioned freezing and number of avoidances and ITCs (see Table 2) give further support to that contention.

In the only previous study with Roman rats in which NH effects were evaluated on shuttle box avoidance, only “outbred” RLA males (from the swiss RLA/Verh outbred line) were used (Escorihuela et al., 1995). This study indicated a slight trend toward a positive treatment effect on avoidance responses, which failed to be significant according to overall ANOVA (Escorihuela et al., 1995). Therefore, the present study in inbred Roman rats of both sexes is the first demonstration of a significant NH-induced modulation of the trait which is the criterion for selection of the RLA-I and RHA-I strains (i.e., shuttle box avoidance acquisition).

Conditioned freezing and two-way avoidance acquisition (as well as ITCs) are apparently less affected by NH than the unconditioned anxiety measures (NOE, ZM). This would be congruent with the view that, in the Roman rat strains, two-way avoidance acquisition and conditioned freezing are more strongly linked to their genetic constitution than unconditioned anxiety/fearfulness traits (e.g., Castanon et al., 1995; Fernández-Teruel et al., 2002b; Steimer and Driscoll, 2003, 2005; Driscoll et al., 2009). Related to that, it was reported already in early behavioral genetic studies in rats that two-way active avoidance acquisition is probably among the types of behavioral traits having the highest heritability coefficients (e.g., Wahlsten, 1972; Wilcock and Fulker, 1973; Wilcock et al., 1981; see also Castanon et al., 1995; Fernández-Teruel et al., 2002b; Johannesson et al., 2009; Baud et al., 2013, 2014). With regard to the Roman rats, it has been suggested that the “warm up” phase, i.e., the performance during initial 10–20 trials of each shuttle box training session, is the aspect that most markedly differentiates both lines/strains (e.g., Driscoll and Bättig, 1982; Fernández-Teruel et al., 1991b; Escorihuela et al., 1995, 1999; Ferré et al., 1995b; Driscoll et al., 2009). In particular, the extremely slow “warm up” effect typically shown by RLA rats seems to stem from their proneness for fear conditioning (e.g., Escorihuela et al., 1995; López-Aumatell et al., 2009a,b; Estanislau et al., 2013), thus to freeze when facing an aversively-conditioned context (as it is the case during the initial trials in the shuttle box task), which is known to run against actively searching for a more adaptive (active) response like escape or avoidance (e.g., Weiss et al., 1968; Wilcock and Fulker, 1973; Fernández-Teruel et al., 1991a,b; Gray and McNaughton, 2000; López-Aumatell et al., 2009a,b; Vicens-Costa et al., 2011; Díaz-Morán et al., 2012). Hence, it seems possible that a more proactive (or less reactive) coping style of NH-treated RLA-I rats (as suggested by NH effects on conditioned freezing and ZM and NOE tests) might be partly responsible for their improved ability to acquire the two-way avoidance task.

As said in the Introduction, some studies on NH that have used rats of both sexes have shown that “treatment × gender” interactions are common, and either NH effects are often observed in just one gender or handling effects show divergent patterns in both sexes. As a few examples of this: (1) Stamatakis et al. (2008) reported that in acutely-stressed (Wistar) males rats NH manipulated showed better place learning performance than females, while no sex differences were observed in a spatial memory trial. (2) Likewise, handling-induced changes in hippocampal mineralocorticoid receptors were found in males only (Stamatakis et al., 2008). (3) Learning of a spatial “Y” maze task was impaired by NH in males and improved in female Wistar rats (Noschang et al., 2012) while, in the same study, (4) only NH-treated females (but not males) showed a decreased SOD/CAT (superoxide dismutase/catalase) ratio in prefrontal cortex. (5) Impairing NH effects on long-term retention of inhibitory avoidance were observed in female, but not male Sprague-Dawley rats (Kosten et al., 2007). (6) In another study, NH produced sex-dependent effects on stress-induced corticosterone and brain *c*-*fos* expression in adolescent Sprague–Dawley rats (Park et al., 2003). (7) Furthermore, Papaioanou et al. (2002) reported that NH treatment interacts with stress type (i.e., short-term or long-term) and with sex to induce changes in the concentration and turnover of brain serotonin and dopamine in Wistar rats. In this context, it is remarkable that also in the present study the positive effects of NH on avoidance acquisition have been shown to be divergent depending on gender. Thus, there are significant “NH × sex” effects on SHAV50 and SHAV100 (avoidances after 50 or 100 trials, respectively), which reflect the fact that NH improved avoidance acquisition more markedly in female rats of both strains during the first 50 trials (SHAV50; see Figure 6B) or in RLA-I females (compared with RLA-I males) after completing the 100 trials (SHAV100; see Figure 6C).

There is evidence, from factor-analytical studies using very large samples of F2 rats (derived from the “outbred” Roman lines, *n* = 800; Aguilar et al., 2003) or heterogeneous NIH-HS rats (*n* = 1600; López-Aumatell et al., 2011) that females' responses when facing conflicting situations might be more driven by activity-related responses (i.e., more “proactive” responses) than males' responses, which would be more driven by anxiety/freezing (i.e., “reactive” coping strategies; e.g., Fernandes et al., 1999; Aguilar et al., 2003; López-Aumatell et al., 2011). In this connection, it is tempting to suggest that the more marked NH effects observed in females, particularly in the two-way avoidance task, might be partly due to the fact that NH is able to disinhibit conflict-induced behavior (i.e., so changing a “reactive” to a more “proactive” coping strategy) more easily in females than in males.

The present positive results of NH on two-way avoidance acquisition are in contrast with several lines of research carried out by using psychogenetically-selected strains/lines of rats possessing divergent abilities to acquire shuttle box avoidance (i.e., Mausdley reactive vs. non-reactive rats, Levine and Broadhurst, 1963; RLA/Lu v.s RHA/Lu rats, Satinder and Hill, 1974), which failed to show acquisition improvements following neonatal handling. Possible reasons to explain the different results of these and the present study could be the more intensive neonatal handling procedure used here (i.e., two handling sessions/day in the present study v/s one session/day in those studies), or the fact that the present shuttle box training parameters (i.e., composite “light + tone” CS; CS, US and inter-trial interval of longer durations than in those studies; no overlapping between CS and US) were specifically selected to facilitate the emergence of escape (or avoidance) responses and to minimize the presence of “response failures” (see details in Escorihuela et al., 1995).

The observed between-strain differences in baseline acoustic startle (BAS) are in agreement with previous reports (e.g., López-Aumatell et al., 2009a,b). Notably, however, neonatal handling did not affect BAS responses in any rat strain. The baseline acoustic startle is a reflex response that is mediated by a fast “cochlear root nucleus—caudal pontine reticular nucleus” pathway (e.g., see review by Koch and Schnitzler, 1997). To the best of our knowledge the effects of NH treatment on BAS have been evaluated for the first time in the present study, and the absence of changes in NH-treated rats, which contrasts with the positive effects observed in the other tests/tasks, suggests that brainstem-mediated reflex responses (i.e., BAS) are less sensitive to (NH) manipulation influences than more cognitively elaborated conflict-based responses (like NOE, ZM, CCF, or SHAV), which are thought to be under hippocampal control (e.g., Gray and McNaughton, 2000; López-Aumatell et al., 2008, 2009a, and references therein). Possibly in line with that contention, in a study in which rats were treated with environmental enrichment (EE) for several months, the treatment produced the expected long-lasting positive effects on several stress/anxiety-related and cognitive responses, but EE did not affect baseline acoustic startle (Peña et al., 2009).

A more active/functional hippocampus has been related to increased anxiety when facing “approach-avoidance” or “passive avoidance/active avoidance” conflict situations (such as the cases of NOE-ZM and CCF tests and the SHAV task, respectively) (Gray and McNaughton, 2000). In line with that, it is remarkable that the high anxious (and passive/reactive coper) RLA-I rat strain has a more functional hippocampus than the (low anxious) RHA-I strain (Meyza et al., 2009; Garcia-Falgueras et al., 2012). It would be interesting to investigate how hippocampal function during (unconditioned or conditioned) conflict could be affected by neonatal handling and how such an effect on hippocampus would be relevant for the H-induced changes in RLA-I rats. Would NH manipulation influence septo-hippocampal function in a manner similar to anxiolytic drugs—i.e., benzodiazepine agonists, which reduce conflict and improve shuttle box avoidance acquisition? (e.g., Fernández-Teruel et al., 1991a; Gray and McNaughton, 2000). A number of effects of neonatal handling on different neurobiogical aspects within the hippocampal formation have been reported, for example: (i) increased hippocampal long-term potentiation (e.g., Wilson et al., 1986) and decreased hippocampal neuronal loss with age in H-treated rats (e.g., Meaney et al., 1988; see reviews by Fernández-Teruel et al., 1997, 2002a); (ii) enhanced hippocampal type II glucocorticoid receptors, linked to decreased HPA-axis responses to stress (e.g., Meaney et al., 1988); (iii) increased GAP-43 (growth associated protein 43) expression in rat pups (Zhang et al., 2012); (iv) increases in hippocampal but not cortical 5-HT and 5-HIAA in rats (e.g., reviewed by Anisman et al., 1998; Fernández-Teruel et al., 2002a), as well as in hippocampal nerve growth factor mRNA (Mohammed et al., 1993; Pham et al., 1997); (v) enhancement of NADPHdiaphorase-positive neurons (a potential marker of nitric oxide-producing neurons) (Vaid et al., 1997); (vi) increases of central benzodiazepine and GABA-A receptors (Bodnoff et al., 1987; Bolden et al., 1990; see review by Raineki et al., 2014). Preliminary results from our laboratory suggest that RLA-I rats have reduced content of hippocampal PSA (polysialic acid, related to neural cell adhesion molecules -NCAM-), which is raised to RHA-I levels by NH. Thus, provided that all these forms (and others not listed here) of hippocampal plasticity have been shown to be sensitive to NH effects, it does not seem unreasonable to expect that hippocampal function during conflict (i.e., under anxiety-inducing, conditioned or unconditioned) situations could also be enduringly modulated by neonatal handling, thus inducing changes on coping strategies/responses. Testing such a hypothesis should be matter of further research.

In summary, in the present study, several long-lasting effects of NH are reported for the first time: (i) NH manipulation is able to partially counteract the genetically-based two-way avoidance acquisition deficit of (inbred) RLA-I rats, being the effect more evident in females. (ii) NH manipulation improves acquisition in females (but not males) of the RHA-I strain. (iii) NH effects on shuttle box avoidance acquisition are paralleled by a treatment-induced reduction of context-conditioned freezing (during inter-trial intervals 2–5 of the training session) also in both rat strains, which may suggest that the treatment has produced some change toward more adaptive (i.e., proactive) coping strategies, and that such an effect may underlie (at least partly) the avoidance acquisition improvement, particularly in RLA-I rats. (iv) The positive effects of NH on SHAV, ITCs, CCF, NOE, and ZM test measures, also agree with the contention that the treatment induces changes toward more proactive coping strategies. (v) Baseline acoustic startle is not influenced by NH, in line with findings obtained with other anxiety-reducing environmental treatments (Peña et al., 2009), thus suggesting that brainstem-mediated responses like BAS could be less sensitive to chronic treatment influences than conflict-based hippocampus-mediated responses.

### Conflict of interest statement

The authors declare that the research was conducted in the absence of any commercial or financial relationships that could be construed as a potential conflict of interest.
